# Few-Shot Conditional Learning: Automatic and Reliable Device Classification for Medical Test Equipment

**DOI:** 10.3390/jimaging10070167

**Published:** 2024-07-13

**Authors:** Eva Pachetti, Giulio Del Corso, Serena Bardelli, Sara Colantonio

**Affiliations:** 1 “Alessandro Faedo” Institute of Information Science and Technologies (ISTI), National Research Council of Italy (CNR), 56127 Pisa, Italy; 2 Department of Information Engineering (DII), University of Pisa, 56122 Pisa, Italy; 3 Centro di Formazione e Simulazione Neonatale (Centro NINA), Azienda Ospedaliero Universitaria Pisana (AOUP), 56126 Pisa, Italy

**Keywords:** few-shot learning, trustworthy AI, uncertainty quantification, simulation-based medical education, newborn resuscitation

## Abstract

The limited availability of specialized image databases (particularly in hospitals, where tools vary between providers) makes it difficult to train deep learning models. This paper presents a few-shot learning methodology that uses a pre-trained ResNet integrated with an encoder as a backbone to encode conditional shape information for the classification of neonatal resuscitation equipment from less than 100 natural images. The model is also strengthened by incorporating a reliability score, which enriches the prediction with an estimation of classification reliability. The model, whose performance is cross-validated, reached a median accuracy performance of over 99% (and a lower limit of 73.4% for the least accurate model/fold) using only 87 meta-training images. During the test phase on complex natural images, performance was slightly degraded due to a sub-optimal segmentation strategy (FastSAM) required to maintain the real-time inference phase (median accuracy 87.25%). This methodology proves to be excellent for applying complex classification models to contexts (such as neonatal resuscitation) that are not available in public databases. Improvements to the automatic segmentation strategy prior to the extraction of conditional information will allow a natural application in simulation and hospital settings.

## 1. Introduction

In many contexts, integrating automatic classification methods that are fast, easy to use, and reliable is increasingly essential. Whether in healthcare, finance, cybersecurity, or customer service, the ability to quickly and accurately categorize data can significantly enhance operational efficiency, decision-making, and overall service quality. Traditional manual classification methods are often time-consuming, resource-intensive, and prone to human error. The latter is particularly critical in the medical field, where a mistake by an expert can have serious consequences. As a result, the demand for automated classification systems that deliver high performance with minimal user intervention is more critical than ever. Recent advances in deep learning have significantly contributed to the development of sophisticated classification techniques [1,2,3,4]. These techniques typically rely on large datasets and advanced computational power to learn intricate patterns and make accurate predictions. However, in many contexts, access to powerful computational resources and large training datasets is not straightforward. When training data are insufficient, models might learn spurious correlations rather than underlying patterns, adversely affecting their generalization performance. In addition, the reliability of the predictions cannot be accurately estimated by most machine learning techniques. Bayesian-style models that estimate a measure of prediction confidence at high computational cost are often used to define an uncertain class [5]. Conversely, the common practice of using the model’s own implied confidence intervals can lead to poorly calibrated confidence measures [6,7] or unreliable results [8,9].

Few-shot learning (FSL) has emerged as a possible solution to address the challenging task of training effective deep learning models in data-scarce scenarios [10,11,12,13,14]. One of the most significant applications of FSL lies in medical image analysis, where privacy issues, high data acquisition costs, and the intensive nature of expert annotation often constrain dataset sizes. Through FSL, models can attain robust generalization from a limited pool of labeled examples, thereby enabling the development of potent medical imaging solutions despite data scarcity. The meta-learning paradigm provides one of the most promising approaches to address FSL tasks. Also known as learning-to-learn, meta-learning enables models to swiftly adapt and generalize across new tasks using minimal training examples. Indeed, unlike traditional training approaches focusing on model training with datasets, here, models are trained on episodes. This exposure allows the model to acquire transferable knowledge and discern spurious patterns. Consequently, when presented with a novel episode in the testing phase, the model can adeptly apply its meta-knowledge to yield accurate predictions despite the example scarcity.

This study explores a few-shot (max five example data per item) automatic classification scenario leveraging a meta-learning paradigm and integrating conditional information (contour of the object) extracted using a state-of-the-art segmentation model. Indeed, in this context, objects possess unique shapes that are pivotal for class differentiation. At the same time, objects may vary in color, texture, and illumination, so leveraging binary masks helps to underscore these shapes, furnishing the model with definitive information on the object’s contours and architecture. We further introduce a post hoc probabilistic reliability score (RS) to couple each deterministic prediction with an estimate of trustworthiness. The obtained model provides a fast and reliable classification of objects from a limited set of images (∼100), allowing the application of this approach to several applicative contexts. In particular, the field of medical education provides an example of practical application. Indeed, it represents a domain based on the use of procedures and equipment that are constantly updated to improve the quality of care. The application example shown in this manuscript is related to neonatal resuscitation. Neonatal resuscitation is a medical intervention to help establish breathing and circulation during the transition from intrauterine to extrauterine life. A critical factor is the availability of appropriate clinical equipment, which is associated with a five times greater likelihood of successful resuscitation. Therefore, the World Health Organization recommends that equipment and supplies for delivery, including neonatal resuscitation, should be available when delivery is planned [15,16].

The use of simulation in neonatal resuscitation training is recommended for healthcare personnel [17] because it has been shown to increase patient safety by reducing the duration of neonatal resuscitation, increasing the effectiveness of teamwork, and improving procedural knowledge [18,19,20] However, to the best of our knowledge, there are no technological aids for the checklist during simulation training, which is covered during the theoretical course and then approached in the pre-simulation briefing with the user familiarizing themselves with the instrumentation provided. This task is complicated by the recent proliferation of equipment brands (both reusable and disposable) [21]; therefore, a machine learning method trained on one specific hospital setting is unlikely to adapt to another. Therefore, this is an optimal context to test a technique based on a reduced dataset of natural images that can be applied without the need for re-training (but only supports replacement), such as FSL with RS.

## 2. Data Acquisition and Preparation

We present the Medical Equipment Dataset, which contains less than 100 natural images of specialized instruments used to tune the network, and the BAIR Robot Pushing dataset [22], which we used to train a network that extracts features from the masks of the equipment under investigation.

### 2.1. Medical Equipment Dataset

**Figure 1:**
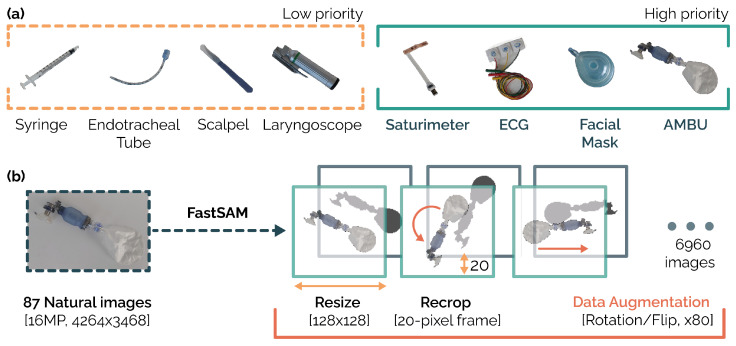
Panel (**a**): medical equipment included in the checklist of the neonatal resuscitation procedure. Panel (**b**): data augmentation and cleaning procedure that generates the meta-training dataset (6960 images) from the original 86 images.

The equipment that was selected for application included in the context of the neonatal resuscitation procedure are all instruments found within the materials checklist given within the “Manual of Neonatal Resuscitation” [23] as equipment that must be readily available and functional for each delivery. Among the materials within the checklist, four higher-priority and four lower-priority instruments were identified. The equipment selected as high priority has been identified according to the latest guidelines, which highlight some important advances in understanding how best to resuscitate newborns, including monitoring techniques to guide resuscitation efforts and control of the normal range of blood oxygen levels. International clinical guidelines and resuscitation algorithms recommend using the newborn infant’s breathing, heart rate, and peripheral oxygen saturation (*SpO2*) to guide the resuscitation process. Neonatal clinicians must therefore be able to quickly and accurately monitor these signs and ensure that normal ranges for these parameters are defined [15]. Based on this principle, the electrodes for ECG (*Philips*) and a saturimeter (*Masimo*) were selected for the assessment of clinical status, while AMBU (*DEAS*) and a facial mask (*size 1, AMBU+*) are needed for the provision of ventilatory assistance; see Figure 1a. The instruments identified as having the lowest priority are tools needed to perform steps in the rarer neonatal resuscitation procedure: the endotracheal intubation procedure in which a laryngoscope (*blade size 1, Macintosh*) and an endotracheal tube (ET, *size 3.5 PORTEX*) are needed, and the drug administration procedure in which a syringe (*0.5 mL CARESS*) and a scalpel (for umbilical stump preparation) are required. Images were captured using the 16MP camera of a Samsung Galaxy a52, acquiring a representation of the instruments under various light sources: natural light, neon light (characteristic of hospital environments), and warm light (characteristic of environments where neonatal resuscitation is performed). Each instrument was photographed while placed on top of a preparatory table with a homogeneous background and with dual perspective views: a top view shot and a “user view” shot (placing the phone at an observer’s eye level).

This dataset (n=87) is not large enough to fine-tune an artificial neural network. Therefore, we performed rigorous data augmentation and cleaning to improve the quality of the information and to artificially expand the dataset in order to avoid model overfitting (see Figure 1b). In particular, we segmented each natural image captured (16 MP, 4264 × 3468) using a state-of-the-art segmenter (FastSAM [24], a less computationally demanding version of Segment Anything Model [25]). An erosion (square kernel, k=5) and dilation (square kernel, k=5) procedure was performed on the resulting masks and the main connected component was retained. The background (defined as what is not contained in the main connected component of the mask) was then removed from the original image. The coupled images (natural image with background removed and associated mask) were then cropped and resized so that they had a 128 × 128 pixels resolution and a frame size of 20 pixels per side. The 174 coupled images thus obtained (87 RGB and 87 masks) were then used to generate the augmented dataset; specifically, they were subjected to a flip (horizontal and vertical, augmentation factor ×4) and rotated with respect to the center of mass of the mask (20 rotations of 18°, augmentation factor ×20), resulting in 13,920 coupled images (6960 RGB and 6960 masks). In order to avoid positively biased results given by the data augmentation procedure, we applied a rigorous stratification to the training/validation/cross-validation procedures. Therefore, if the original image was assigned to a particular set (e.g., training/validation/test), each image generated by the data augmentation was assigned to the same set, preventing data leakage.

### 2.2. Model Equipment Test Dataset

To create a completely unbiased test set, we set up 10 different preparatory tables with the eight available medical tools arranged in a random configuration as the default natural application setting. The images, including the entire preparatory table, have a larger field of view and, therefore, each object was encoded with a reduced resolution (especially small devices such as ET or scalpel).

### 2.3. BAIR Robot Pushing Dataset

**Figure 2:**
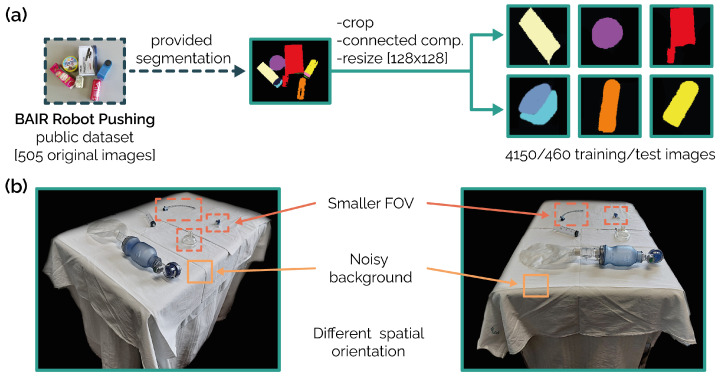
Panel (**a**): BAIR Robot Pushing public dataset preprocessing (505 original images to 4610 masks). The procedure includes isolating a single connected component, central cropping around the mask, and nearest neighbor resizing [128 × 128 pixels]. Panel (**b**): examples of two real-world scenarios used as a test set.

To extract shape information (using the encoding part of an ad hoc-trained autoencoder), we used object annotations from the BAIR Robot Pushing dataset as a training set [22]. Focusing on training image annotations, we employed a multi-step preprocessing pipeline to isolate single object masks (see Figure 2a). First, object separation based on pixel values within the mask divided the scene. Next, the connected-component analysis further refined the individual object masks. We then identified the centroid of each mask and cropped the image around it to focus on the single object. Finally, we resized all the images to a uniform size of 128 × 128 pixels. This process resulted in 4610 masks, divided into training (4150) and test (460) sets.

## 3. Materials and Methods

### 3.1. Few-Shot Problem Definition

In this work, we addressed an equipment object classification task in a low-data scenario by leveraging a meta-learning framework to train a base learner. During the training phase, also known as *meta-training*, we sampled a set of episodes from a given dataset $D_{base}$. Each episode consisted of a *support* set $S={\left\{\left(x_{i},y_{i}\right)\right\}}_{i=1}^{N}$ and a *query* set Q = ${\left\{\left({\hat{x}}_{i},{\hat{y}}_{i}\right)\right\}}_{i=1}^{M}$, where $x_{i}$ represents an image sample and $y_{i}$ is its corresponding label. The total number of support samples *N* is given by N=K·H, where *K* is the number of classes in each episode and *H* is the number of data samples for each class. The total number of query samples *M* is given by M=K·J, where *J* represents the number of query images for each class. During meta-training, the goal was to minimize a loss function L on the query samples, conditioned on the support set. In the meta-validation stage, several episodes were sampled as well. In this phase, the base learner was provided with previously unseen episodes and performed each episode’s classification task by leveraging the meta-knowledge learned during the meta-training phase.

We employed the Prototypical Network (ProtoNet) proposed by Snell et al. [26] as our meta-learning framework. Here, the meta-trained base learner extracted feature embeddings from all the support and query samples in each episode. The support embeddings of each class were then used to compute the class prototype by averaging all the support embeddings belonging to that class. The final classification was done by measuring the Euclidean distance between the query embeddings and all class prototypes. The query sample was classified as belonging to the class to whose prototype it is closest in feature space. In the next section, we describe our proposal in detail.

### 3.2. Proposal

Our proposed approach is designed to perform equipment object classification in an extremely low-data scenario. To address this challenge, we employed three main strategies: an intense data augmentation to increase the dataset size, a multi-branch model to provide shape-conditional information, and a prototypical meta-learning framework, a popular method for tackling FSL, to enhance the model’s generalization capabilities. In the following, we describe our approach in detail.

**Figure 3:**
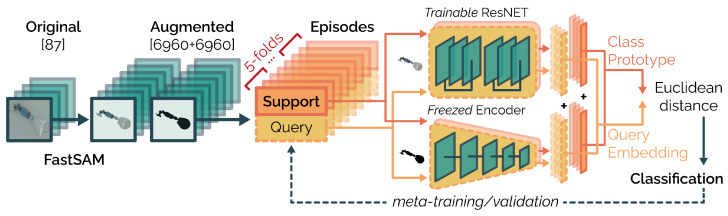
Illustration of our proposed approach. Starting with our original dataset, we use the FastSAM model to isolate objects in the images. We then apply extensive data augmentation to both the masked images and their binary masks, increasing the dataset size by 80 times. The augmented dataset is split into training and validation sets using 5-fold cross-validation. For each training/validation set, we create several meta-training and meta-validation episodes to perform prototypical meta-training. Specifically, for each episode, we provide the support and query masked images to the ResNet base learner and the corresponding binary masks to the pre-trained encoder with frozen parameters. We concatenate the output features from the two models for each support and query sample. The class prototype is calculated by averaging the features of the support samples belonging to the same class. Finally, classification is performed by measuring the Euclidean distance between each class prototype and each query embedding. This procedure is repeated for each meta-training and meta-validation episode.

Starting from a set of object images, we first used FastSAM [24] to extract the object masks. We then applied several data augmentation techniques to both the masked images and their binary masks, increasing the size of the dataset by a factor of 80. We then split the augmented dataset into meta-training and meta-validation sets according to a 5-fold cross-validation (CV) and derived the corresponding meta-training and meta-validation episodes, each consisting of a support and a query set. Unlike the canonical approach, where each support and query sample contains an image and its corresponding label, here, each sample also contained the corresponding binary object mask as well. For each episode, the masked images were provided to the base learner, which, in this case, was a ResNet model. Simultaneously, the binary masks were fed to a pre-trained encoder with frozen parameters. The ResNet extracted an embedding feature vector from each support and query image, while the encoder extracted features from the corresponding binary masks. This multi-branch approach prioritized the extraction of shape features along with other features typically extracted from the image, as shape is a paramount feature in this task. Later, the features extracted by the ResNet and by the encoder were then concatenated into a single embedding for each support and query sample. All the support embeddings of the same class were then averaged to compute the class prototype. Finally, the Euclidean distance between each class prototype and the query embeddings was computed for classification. We provide a representation of our proposed approach in Figure 3.

After completing the meta-training phase, we assessed the model’s performance on a separate test set that underwent the same preprocessing steps as the meta-training/validation data. In anticipation of a real-world application where it is necessary to evaluate the model’s performance in classifying a specific object at a time rather than within a generic classification episode, we chose to perform inference using a classical approach instead of an episodic approach. To achieve this, we randomly selected a set of support samples, which we used consistently throughout the inference phase. During inference, the support samples were provided to the trained multi-branch model to extract features from both the image and its mask, as done during the meta-training/validation phase. Next, the single test image to be classified was provided to the model to extract its feature embedding. Classification was then performed as previously described, using the Euclidean distance between the test image’s features and the class prototypes derived from the support samples.

In addition to the predicted class, we also provided a reliability score as output during the inference phase. The score was computed by adapting a classical post hoc reliability metric (trust score, [27]) to the structure of a few-shot learning model. In particular, we took advantage of the features naturally used to compute the Euclidean distance to classify the query elements to define a normalized version of the reliability score on a simplified version of the feature space. This allowed a prediction to be coupled with an estimate of the reliability of the model output.

### 3.3. Define a Reliability Score

Defining when a model is uncertain about a prediction is critical to being able to give the user the confidence to trust an automated machine learning tool. Careful model design and large amounts of data are required to train many of the techniques used to establish credibility scores, such as Bayesian Neural Networks, Credibility Networks, or Deep Ensemble [5]. Alternatively, internal model scores are commonly used as a non-intrusive/post hoc technique to produce a reliability score but are known to be poorly calibrated [6,7] and overconfident [8,9]. An alternative non-intrusive approach was introduced by Jiang et al. under the name trust score [27].

Formally, given a test sample *x*, a classifier M (with a set of possible classes K), and a highly representative subset $H_{\alpha }\left(K_{i}\right)$ of the training set for each given class $K_{i}$ (the so-called α-high-density set of the class $K_{i}$), the trust score is defined as the ratio between the distance from the testing sample to the α-high-density-set of the nearest class different from the predicted class, and the distance from the test sample to the α-high-density-set of the class predicted by the classifier M:

(1) $$\left\{\begin{array}{c} H_{\alpha }\left(K_{i}\right):=\alpha \text{-set}\left(\{\left(x_{i},y_{i}\right)\in X\times Y|y_{i}\in K_{i}\}\right)\\ \hat{h}\left(x\right):={\mathrm{argmin}}_{K_{i}\in K|K_{i}\neq M\left(x\right)}d\left(x,H_{\alpha }\left(K_{i}\right)\right)\\ \mathrm{TS}\left(x\right):=\frac{d\left(x,H_{\alpha }\left(\hat{h}\left(x\right)\right)\right)}{d\left(x,H_{\alpha }\left(M(x)\right)\right)}\in \left[0,\infty \right) \end{array}\right.$$

where $\tilde{h}\left(x\right)$ is the second optimal closest class, *d* is an arbitrary distance (Euclidean k-nearest neighbor, distance from the centroid, etc.) computed on the last dense layer of the artificial neural network, and TS(x) is the trust score of the image *x*. Therefore, the main elements of the trust score are a small (informative) set of images of each class (the α-high-density set of the class $K_{i}$), a simplified model (k-cluster classifier based on Euclidean distance of a given feature space) used to overcome the overspecialization of the original classifier M, and an appropriate choice of a layer to use for extracting a feature representation to compute the distance. It can be shown that for labeled data distributions with well-behaved class boundaries, the classifier is likely to agree with the Bayes-optimal classifier when the trust score is large [27]. However, the reliability measure obtained is less informative for high-dimensional feature spaces (such as those obtained by computation on ViT or VGGs), and the fact that the trust score has no upper bounds leads to a difficult interpretation of the results.

Specializing this formula for the few-shot framework is straightforward. In fact, there is no need to define an α-high-density set, since the support used to produce the prototype encodes complete (and high-density) information about the feature space. The feature space coincides with the feature representation used to compute the Euclidean distance between the prototype and the query, so the formula simplifies to the ratio between the second and first classification classes. For few-shot learning approaches, since the classification coincides with the minimum distance on the feature space, it holds that $d\left(x,H_{\alpha }\left(M(x)\right)\right)\leq d\left(x,H_{\alpha }\left(\hat{h}\left(x\right)\right)\right)\in \left[0,\infty \right)$ and, therefore, ${\mathrm{TS}}^{-1}\left(x\right)\in \left[0,1\right]$, where 0 is an optimal prediction (*x* matches with the class prototype) and 1 is the worst-case scenario (*x* is halfway between two different prototypes).

**Figure 4:**
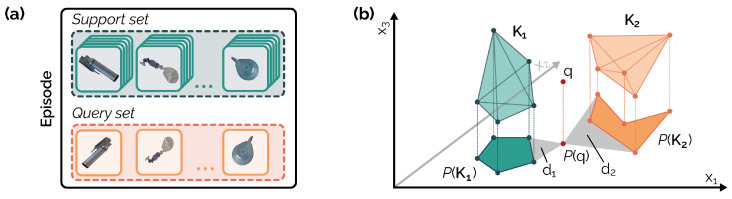
Panel (**a**): example of an episode containing a support set (i.e., 1–5 images for each class) and a query set (1 image to be classified for each class). During meta-training, the distance between each query element and the support set is computed to define the predicted class. Panel (**b**): projection of a high-dimensional feature space to a lower-dimensional one using a model reduction technique. A reliability score is calculated as the ratio of the distances between the convex hull of the projected cluster of the predicted class and the second optimal classification cluster.

However, to avoid overconfident results due to using a function of the classification metric as the reliability score [8] and the risk of using a high-dimensional feature space to compute the trust score [27], we need a simplification of the classification model M. Therefore, given the support and the query *x* that we want to classify, we performed a feature reduction using an exact Principal Component Analysis retaining 95 of the variance of the model (see Figure 4, the projection of the original). More advanced non-linear dimensionality reduction methods such as ISOmap [28], Umap [29] or VAEs [30] can be used to define a low-dimensional feature space. However, due to the extremely small size of the elements to be used for dimensionality (∼5, in a few-shot fashion), we decided to use an elementary linear reduction approach and investigate modern alternatives in future work. The exact PCA approach leads to a feature space of size $\leq \min\left(\#\mathrm{features},\#K\cdot \#\mathrm{shot}+1\right)$ (in this case ≤40). Furthermore, instead of computing the distance by comparing only the centroid of the 5-shot evaluation (the prototype centroid), we computed the score on the convex hull of the provided 5 elements of the prototype, thus smoothing the prediction and reducing the complexity of the provided model. The reliability score provided is therefore defined as

(2) $$\left\{\begin{array}{c} H_{\alpha }\left(K_{i}\right):=\text{5-Shot-Support}\left(K_{i}\right)\\ \hat{h}\left(x\right):={\mathrm{argmin}}_{K_{i}\in K|K_{i}\neq M\left(x\right)}d\left(x,H_{\alpha }\left(K_{i}\right)\right)\\ \mathrm{RS}\left(x\right):={\;\int \int \;}_{\mathrm{CH}\left(M\left(Px\right)\right)),\mathrm{CH}\left(\hat{h}\left(P\left(x\right)\right)\right)}\frac{d\left(\tilde{x},H_{\alpha }\left(M(\tilde{x})\right)\right)}{d\left(\tilde{x},H_{\alpha }\left(\hat{h}\left(\tilde{x}\right)\right)\right)}d\tilde{x}\in \left[0,1\right] \end{array}\right.$$

where $CH(K_{i})$ is the convex hull of the class $K_{i}$ and P is the projection on the low-dimensional feature space. Intuitively, the reliability score is the average distance computed between a random combination of the convex hulls of the projection on a low-dimensional space of the original support point of the candidate class and the second-best candidate class. It should be noted that this integral can be evaluated as a forward Uncertainty Quantification problem [31] on a low-dimensional feature space (those obtained during the PCA reduction, i.e., ≤41), and, therefore, an efficient sampling strategy can be used to evaluate it (Latin Hypercube Sampling combined with an adaptive halting criterion [32]).

### 3.4. Experiments

#### 3.4.1. ResNet

As our base learner for meta-learning, we used a ResNet model, evaluating both ResNet-18 and ResNet-50 architectures. Given our limited data and the high computational effort required to train the numerous parameters of a ResNet, we opted to use pre-trained parameters from the ImageNet dataset. These pre-trained parameters provide a solid starting point, enabling the network to converge faster during training and to leverage already-learned features for the new task. This approach improves the model’s overall accuracy and robustness while significantly reducing the computational resources and time required for training. During training, we used a learning rate of $10^{-3}$, a weight decay of $10^{-5}$, and trained for 50 epochs. We employed Cross Entropy loss as the function to be optimized and Stochastic Gradient Descent as the optimization method. Additionally, we used a learning rate decay scheduler to reduce the learning rate by a factor of 0.1 after 30 epochs.

#### 3.4.2. Masks Encoder

We developed and trained an encoder architecture to utilize object segmentation masks generated by the segmentation model as an additional information source for improved object classification. The goal was to leverage the autoencoder to extract meaningful features from the object masks and combine them with features extracted from the images. We built the autoencoder from scratch, performing a grid search to obtain the best-performing architecture [33]. We evaluated three to five convolutional blocks in both the encoder and decoder and whether to leverage max-pooling layers after each convolutional layer or not. We utilized a $10^{-3}$ learning rate, $10^{-5}$ weight decay, a batch size of 20, and 50 epochs for training. We also employed the Mean Squared Error (MSE) loss function and the Adam optimizer. We evaluated the structural similarity index (SSIM) [34] between the original and reconstructed masks to assess our model’s performance. Additionally, since the shape of the reconstructed mask is of primary interest, we also evaluated the Dice score between the original and binarized reconstructed masks.

#### 3.4.3. Few-Shot Experiments

We conducted several few-shot experiments using both ResNet-18 and ResNet-50 as base learners, evaluating performance in 1-shot, 3-shot, and 5-shot settings. This means we had one, three, or five sample images per class in the support set. In all our experiments, we included one query image per class to evaluate the model’s performance within each episode. During each epoch, we performed 50 meta-training episodes and 50 meta-validation episodes.

#### 3.4.4. Inference

We evaluated our model’s performance in each episode during meta-training by calculating the mean accuracy and its standard deviation (STD) across all meta-validation episodes. To assess overall performance across the CV folds, we also calculated the median and interquartile range (IQR) across the five folds. For the final inference on the test set, we conducted two different types of evaluations. First, we performed an object-by-object evaluation, assessing metrics such as accuracy, Area Under the Receiver Operating Characteristic Curve (AUROC), Area Under the Precision–Recall Curve (AUPRC), recall, along with the reliability score (RS). Additionally, we conducted a by-table evaluation on the test set, measuring the mean accuracy by table in each fold, along with the median and IQR accuracy across folds. This approach helped us assess the impact of different work table settings on classification performance.

## 4. Results

### 4.1. Masks Encoder Pre-Training

The best-performing architecture contained three convolutional blocks in the encoder and three corresponding transposed convolutional (deconvolutional) blocks in the decoder, without max-pooling. This autoencoder architecture, provided an SSIM of 0.983(0.005) and a Dice score of 0.935(0.044) on the internal test set (BAIR Robot Pushing dataset), while, on the external test set (masks of Model Equipment Dataset), it provided a 0.961(0.008) SSIM and 0.991(0.005) Dice score. We summarized all the results of the autoencoder experiments in Table 1.

### 4.2. Meta-Validation Results

We provide the results of our experiments, showing the mean and STD of accuracy across the meta-validation episodes for each CV fold in Table 2. As expected, increasing the number of samples per class in the support set, i.e., the number of shots, enhances performance for both base learners. Notably, even with just one sample per class in each episode (one-shot), we achieve remarkable results. In the one-shot setting, ResNet-18 achieves a median accuracy of 96.65% across the five folds, with an IQR of 9.05, while ResNet-50 achieves a median accuracy of 89.25%, with an IQR of 8.10.

In all three k-shot settings, ResNet-18 outperformed ResNet-50. Given the limited amount of data, the higher number of parameters in ResNet-50 may have led to slight overfitting. Overall, the best-performing model was ResNet-18 meta-trained in a five-shot setting, achieving a median accuracy of 99.70% with an IQR of 11.5. We evaluated this trained model on the test set, the results of which we provide in the following section.

### 4.3. Details on Equipment and Reliability

**Figure 5:**
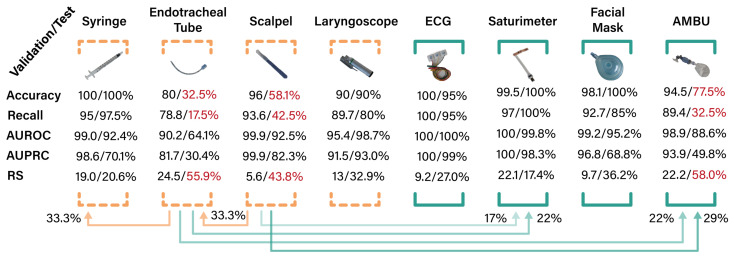
Single class metrics for each device (calculated as the performance to classify that device against all others). Each metric is reported for both the validation set and the test set. The average reliability score (RS) is provided for each class. The arrows below the metrics show (for the test set) the probability of being classified in a different class for incorrect predictions.

To detail the performance of the selected best model and understand the relationship between item classification, we provided a one-at-a-time analysis for each of the medical equipment devices. In particular, as reported in Figure 5, we calculated accuracy/recall/AUROC/AUPRC, and the reliability score (RS). Each device had the same number of images in the test set, so the metrics did not require normalization.

**Figure 6:**
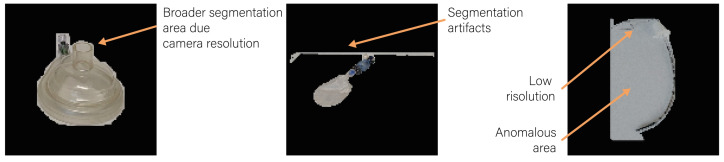
Examples of difficulties induced by a single image containing all the equipment (i.e., preparatory table test set). In particular, segmentation errors and artifacts caused by the low resolution of smaller objects can drastically reduce the performance of the classifier.

The metrics show a very different behavior on the available equipment. In fact, whereas easily recognizable items, such as the syringe, laryngoscope, ECG, facial mask, and saturimeter have an almost perfect model response (test accuracy between 90% and 100%), items with a similar shape (scalpel and ET) have a lower performance, which worsens in the test set. In fact, the ET is often misclassified as a saturimeter, AMBU, or syringe, while the scalpel is often misclassified as an ET or AMBU. For these devices in particular, there is a drop in performance in the test set (i.e., natural images of a preparatory table with medical devices). However, this highlights the difference between a realistic setting with controlled conditions (e.g., cross-validation accuracy of 80% for ET) and a single-photo setting with a variety of different devices (e.g., test accuracy of 32.5% for ET). In fact, as reported in Figure 6, the images collected from the preparatory table have a broader segmentation due to the camera resolution, may contain segmentation artifacts caused by the environment, and, especially for small objects such as ET and the scalpel, the segmentation model may contain incorrect segmentation masks.

The reliability score proves to be efficient in identifying unreliable classes (ET, scalpel and AMBU are the only ones with an RS above 40%). Furthermore, on both the validation and test sets, the score is shown to monotonically follow the metrics of the model.

### 4.4. Test Results by Preparatory Table

As an additional evaluation of our best-performing model, we assessed the test set accuracy on a table-by-table basis for each of the five folds, providing the median and IQR accuracy across the folds. We present the by-table results in Table 3. Our results show that different folds within each table setting behave similarly, resulting in comparable accuracies. This consistency may be attributed to the limited number of data points within each table (one sample object per preparatory table). Overall, if we look at the median accuracy across all folds, we find a best median accuracy of 100% [12.5] and a worst median accuracy of 62.5% [0].

## 5. Discussion and Conclusions

In this work, we tackled an equipment object classification task in a severely data-scarce context. After isolating the objects in the images using the FastSAM model, we employed three main strategies: extensive data augmentation, a multi-branch model, and a prototypical meta-learning framework. The first strategy significantly increased the dataset size by 80 times (from 87 to 6960 images). The multi-branch model, consisting of a ResNet and a pre-trained encoder, enhanced the extraction of shape features, which is crucial for this classification task. Specifically, we pre-trained an autoencoder model and utilized the encoder part to encode the binary masks provided by the FastSAM model. This allowed us to obtain shape-related features, which we concatenated with the features extracted from the masked images by the ResNet. Finally, we employed an episodic training approach using the ProtoNet framework proposed by Snell et al. [26]. This approach helped our model extract the most meaningful features and generalize effectively despite the limited data available.

We conducted our experiments using two base learners (ResNet-18 and ResNet-50) in one-, three-, and five-shot settings. We performed a five-fold CV for each setting to reduce bias and selected the best model based on the highest median accuracy across the folds on the meta-validation set. Our results indicate that the best-performing model is the ResNet-18 trained in a five-shot setting, achieving a median accuracy of 99.70% with an IQR of 11.5. As expected, having more examples per class in the support set led to more meaningful prototypes and, consequently, better classification performance. Nevertheless, even in a one-shot setting, we achieved impressive results, with the ResNet-18 obtaining a median accuracy of 96.65% (IQR 9.05) and the ResNet-50 achieving a median accuracy of 89.25% (IQR 8.10).

We evaluated our best-performing model on the test set using two different approaches. To anticipate real-world applications, we assessed accuracy, recall, AUROC, and AUPRC, along with the reliability score (RS) for each object individually rather than within classification episodes. This approach helped us identify the best- and worst-classified objects and investigate the most frequently misclassified objects, highlighting areas for improvement. Our results, shown in Figure 5, indicate that our model often misclassifies the endotracheal tube, followed by the scalpel and the AMBU. Additionally, we found that the scalpel is frequently misclassified as the endotracheal tube, and the endotracheal tube is misclassified as the syringe. These misclassifications could be attributed to the small size of the objects and their less-recognizable shapes. Furthermore, to evaluate how the preparatory table context affects classification performance, we calculated the accuracy for each table on the test set. We found that preparatory Table 7 provides the best performance in terms of median accuracy across the five folds, achieving 100% with an IQR of 12.5. Overall, our results demonstrate that our model can effectively address an equipment object classification task with competitive results even in an extremely low-data scenario.

The main limitation of this work regards the gap between single-equipment-images (as the training/validation set) and the images containing the entire equipment table. Indeed, increasing the field of view without improving the resolution leads to numerous resolution artifacts that greatly affect model performance. In addition, while the real-time segmentation model used (FastSAM) proves to be efficient in providing correct masks for single device images, the smaller devices are difficult to segment correctly in full table images. It should be noted that the reliability score, even if it provides a good metric for model evaluation, has a high variability due to the small size of the support set (i.e., five elements for five-shot learning). Therefore, it can be used to understand the most reliable/unreliable class, but when used to classify the reliability of a single inference prediction, it tends to mark too many items as “unreliable”.

Future works will focus on reducing these limitations, in particular, using the most complex conditional segmentation model [25] and improving the resolution of the full table acquisition. The detailed one-at-a-time analysis performed allows to understand the need to integrate simple heuristics based on mask size and/or colors, which can provide a final layer of the classification process to avoid AMBU vs. ET/scalpel misclassifications. Further methodological improvements will focus on the integration of an appropriate resampling strategy [31,32] of the support set (which can be randomly increased by random rotation, as shown for the meta-training phase), allowing to produce a more stable ensemble prediction [35]. We will also try different non-linear dimensionality reduction approaches (such as ISOmap [28], Umap [29], or a combination of PCA followed by ISOmap) to see if a geometry-preserving dimensionality method will harm the model reliability score (i.e., by providing overconfident predictions) or, conversely, if it can improve the ability to discriminate reliable/unreliable results. Additionally, we will explore how utilizing advanced meta-learning frameworks that extract more meaningful feature relationships, such as a covariance matrix [36] or Brownian distance covariance [37] or employing a learnable distance metric, could enhance our classification performance.

In conclusion, the results of this study show a promising tool for use in medical training [17,20], which can be applied to different medical settings/equipment without the need for retraining. The development of a system to help identify the correct equipment to prepare can be used in simulation training for many medical procedures where a preparation checklist is used to speed up and improve patient care. Future developments of this project will include the integration of such software on augmented reality hardware, which will make it possible to provide a stable, real-time aid for use in medical training courses, especially for training courses for personnel not specialized in the care of the critical newborn and/or for personnel working in small centers where such events are infrequent.

## Figures and Tables

**Table 1:** Results of the autoencoder in terms of mean and STD. SSIM and Dice score on the reconstructed images on both internal (BAIR Robot Pushing dataset) and external (masks of Model Equipment Dataset) test sets.

Test Set	SSIM	Dice
Internal (BAIR)	0.983 (0.005)	0.935 (0.044)
External (Medical Equipment)	0.961 (0.008)	0.991 (0.005)

**Table 2:** Results for ResNet-18 and ResNet-50 backbones in a *k*-shot setting with *k* ∈ {1,3,5}. The table shows the mean and STD of accuracy across 50 episodes on the meta-validation set for each CV fold. Additionally, it provides the median and IQR of accuracy across the five CV folds. STD: standard deviation; IQR: interquartile range; CV: cross-validation.

Backbone	CV Fold	1-Shot	3-Shot	5-Shot
ResNet-18	Fold 1	89.25 (4.28)	81.60 (5.02)	88.50 (4.72)
	Fold 2	73.40 (6.81)	80.55 (4.98)	77.80 (5.07)
	Fold 3	96.65 (3.59)	100.00 (0.00)	100.00 (0.00)
	Fold 4	98.30 (2.47)	97.55 (2.67)	100.00 (0.00)
	Fold 5	99.40 (1.28)	99.55 (0.96)	99.70 (0.81)
	*Median [IQR]*	*96.65 [9.05]*	*97.55 [17.95]*	*99.70 [11.5]*
ResNet-50	Fold 1	92.80 (3.83)	89.15 (4.02)	91.25 (4.01)
	Fold 2	76.55 (6.50)	77.45 (5.88)	79.40 (6.01)
	Fold 3	98.45 (2.05)	100.00 (0.00)	100.00 (0.00)
	Fold 4	89.25 (5.79)	99.90 (0.49)	99.50 (1.12)
	Fold 5	84.70 (7.79)	96.75 (2.93)	98.75 (1.68)
	*Median [IQR]*	*89.25 [8.10]*	*96.75 [10.75]*	*98.75 [8.25]*

**Table 3:** Table-by-table results for the best-performing model on the test set for each CV fold. The table presents the accuracy for each preparatory table setting, along with the median and interquartile range (IQR) across the five folds for each preparatory table. IQR: interquartile range; CV: cross-validation.

CV Fold	Table 1	Table 2	Table 3	Table 4	Table 5	Table 6	Table 7	Table 8
Fold 1	87.50	75.00	75.00	75.00	87.50	87.50	87.50	87.50
Fold 2	50.00	75.00	62.50	62.50	87.50	75.00	87.50	75.00
Fold 3	75.00	75.00	87.50	50.00	87.50	87.50	100.00	100.00
Fold 4	75.00	87.50	87.50	62.50	87.50	87.50	100.00	87.50
Fold 5	87.50	75.00	87.50	62.50	87.50	87.50	100.00	100.00
*Median [IQR]*	75.00 [12.50]	75.00 [0.00]	87.00 [12.50]	62.5 [0.00]	87.50 [0.00]	87.50 [0.00]	100.00 [12.50]	87.50 [12.50]

## Data Availability

The raw data supporting the conclusions of this article will be made available by the authors on request.

## Abbreviations

The following abbreviations are used in this manuscript:

FSL	Few-shot learning
STD	Standard deviation
IQR	Interquartile range
CV	Cross-validation
RS	Reliability score
TS	Trust score
ET	Endotracheal Tube
AUROC	Area Under the Receiver Operating Characteristic Curve
AUPRC	Area Under the Precision–Recall Curve
